# Nucleolin and ErbB2 inhibition reduces tumorigenicity of ErbB2-positive breast cancer

**DOI:** 10.1038/s41419-017-0067-7

**Published:** 2018-01-19

**Authors:** Eya Wolfson, Shira Solomon, Eran Schmukler, Yona Goldshmit, Ronit Pinkas-Kramarski

**Affiliations:** Department of Neurobiology, Faculty of Life Science, Tel-Aviv University, Ramat-Aviv, 69978 Israel

## Abstract

ErbB2, a member of the ErbB family of receptor tyrosine kinases, is an essential player in the cell’s growth and proliferation signaling pathways. Amplification or overexpression of ErbB2 is observed in ∼30% of breast cancer patients, and often drives cellular transformation and cancer development. Recently, we have shown that ErbB2 interacts with the nuclear-cytoplasmic shuttling protein nucleolin, an interaction which enhances cell transformation in vitro, and increases mortality risk and disease progression rate in human breast cancer patients. Given these results, and since acquired resistance to anti-ErbB2-targeted therapy is a major obstacle in treatment of breast cancer, we have examined the therapeutic potential of targeting the ErbB2–nucleolin complex. The effect of the nucleolin-specific inhibitor GroA (AS1411) on ErbB2-positive breast cancer was tested in vivo, in a mouse xenograft model for breast cancer; as well as in vitro, alone and in combination with the ErbB2 kinase-inhibitor tyrphostin AG-825. Here, we show that in vivo treatment of ErbB2-positive breast tumor xenografts with GroA reduces tumor size and leads to decreased ErbB2-mediated signaling. Moreover, we found that co-treatment of breast cancer cell lines with GroA and the ErbB2 kinase-inhibitor tyrphostin AG-825 enhances the anti-cancer effects exerted by GroA alone in terms of cell viability, mortality, migration, and invasiveness. We, therefore, suggest a novel therapeutic approach, consisting of combined inhibition of ErbB2 and nucleolin, which has the potential to improve breast cancer treatment efficacy.

## Introduction

The four members of the ErbB tyrosine kinase receptor (RTK) family, ErbB1 (EGFR/HER1), ErbB2 (HER2/neu), ErbB3 (HER3), and ErbB4 (HER4), are cell surface receptors, involved in cell proliferation, survival, and growth signaling. Apart from ErbB2, which is an orphan receptor, the ErbBs are activated following ligand binding, which leads to receptor dimerization, and trans-auto-phosphorylation of tyrosine residues in their cytoplasmic tails^1^. Despite being an orphan receptor, ErbB2 is the preferred dimerization partner among its family members, and its association with other ErbBs enhances signaling intensity and dimer stability^2,3^. Hence, not surprisingly, ErbB2 overexpression and amplification are common in various malignancies, especially in breast cancer, where such abnormalities are found in ∼30% of cases^3–5^.

Previously, we have shown that all ErbB receptors functionally bind nucleolin^6^. Nucleolin is a conserved eukaryotic nucleolar protein, which constitutes a vital part of the cell’s growth and survival machinery. In the nucleus, nucleolin participates in many processes, including pre-rRNA transcription and processing, ribosomal assembly and miRNA microprocessing, acts as a helicase, is capable of binding telomerase and topoisomerase I, and mediates cellular stress response through interaction with Hdm2^7–12^. However, the involvement of nucleolin in cell signaling and proliferation is not limited to its nuclear roles, as it shuttles between the nucleus, the cytoplasm and the plasma membrane, and has a wide range of cytoplasmic and membrane activities. Among the reported functions of non-nuclear nucleolin, are binding and stabilization of anti-apoptotic genes mRNA, such as bcl-2, participation in TCR signaling in T-cells and mediation of intracellular import of various proteins, such as heparin-bound growth factors^10,13–17^. Consequently, nucleolin is often involved in tumorigenic transformation and cancer development, and the levels of cell-surface nucleolin in numerous cancer cells are elevated^18,19^.

Recently, we have reported that the physical interaction between nucleolin and ErbB2 triggers activation of the receptor and its downstream MAPK signaling^20^. These are accompanied by increased colony formation and anchorage-independent growth of cells overexpressing both proteins. Moreover, by analyzing data from breast cancer patients, obtained from the Cancer Genome Atlas (TCGA) network, we have found that patients who present with both nucleolin- and ErbB2-positive tumors are at greater disease risk and exhibit lower survival rates compared to ErbB2-positive patients. Importantly, we have found that treatment with the anti-nucleolin G-rich oligonucleotide GroA (AS1411) significantly inhibited the viability and growth of ErbB2-positive breast cancer cells in vitro^20^. Nonetheless, the full scope of GroA treatment in breast cancer, alone and in combination with ErbB2 inhibition, is yet to be examined.

In the present study, we demonstrate that GroA inhibits the activation of ErbB2 in breast cancer xenografts, and markedly impairs growth of breast cancer tumors in vivo. In addition, co-treatment of breast cancer cells with GroA and tyrphostin AG-825, a specific ErbB2 inhibitor^17^, has led to decreased cell viability, inhibition of ErbB2-mediated signaling, increased cell death and, most importantly, suppression of cell tumorigenicity. We, thus, propose GroA as a promising candidate for breast cancer treatment, and pinpoint the ErbB2–nucleolin interaction as a novel target for further development of anti-cancer therapeutics.

## Results

### Nucleolin overexpression enhances in vivo growth of ErbB2-positive breast cancer xenografts

**Fig. 1:**
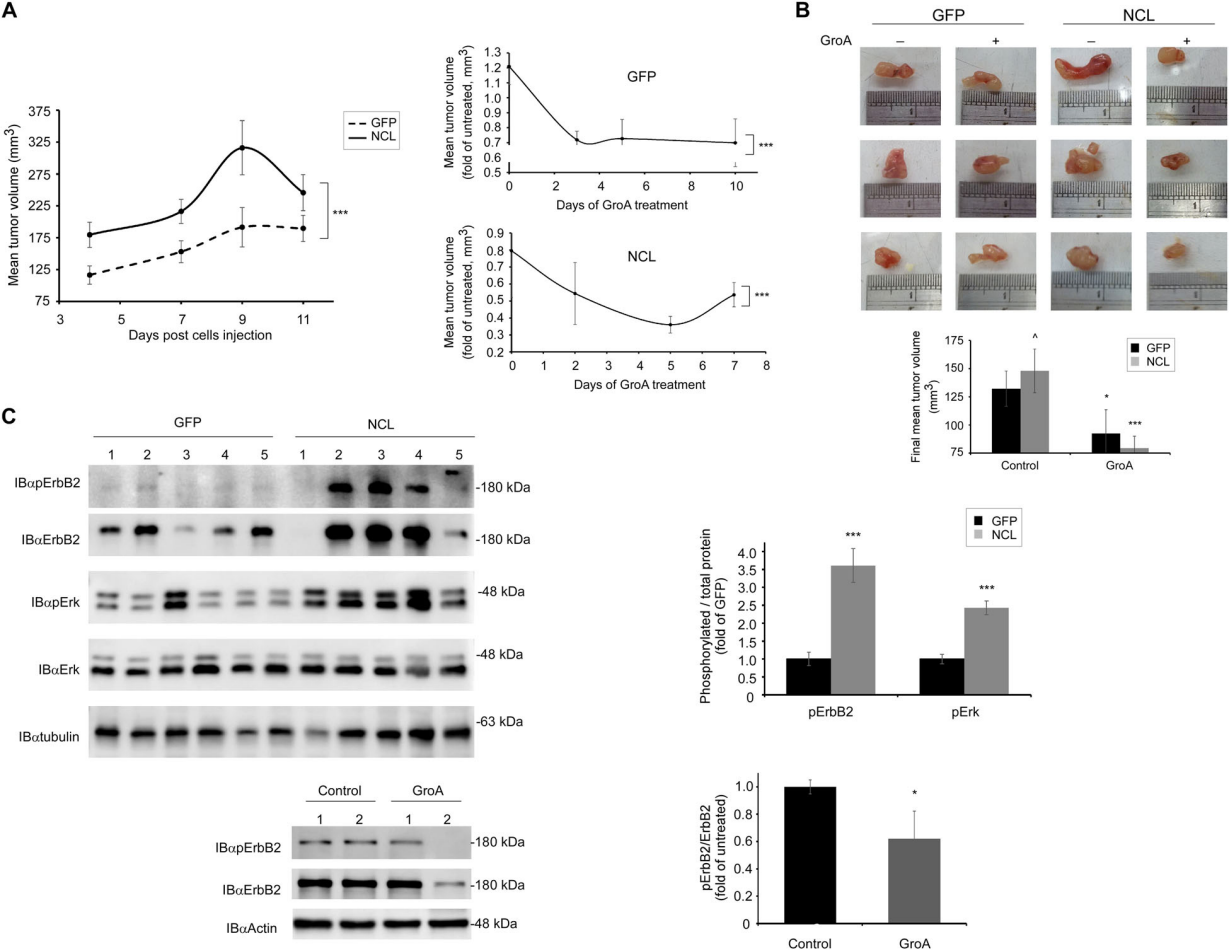
Nucleolin affects growth and development of ErbB2-positive breast tumors in mice **a** Left, comparison of the growth rates and volumes of SKBR3 tumor xenografts, expressing either GFP or GFP-nucleolin (GFP and NCL, respectively; means ± SE; ***p < 0.005; *n* = 5). Right, changes in volume of either GFP or NCL tumors following treatment with the nucleolin-specific inhibitor GroA (AS1411) (means ± SE; ***p < 0.005—compared to the respective untreated tumors; *n* = 5). **b** Upon experiment termination (~20 days), GFP and NCL tumors, either untreated or treated with GroA, were dissected and compared in size. Lower panel, final tumor volumes are presented as means ± SE (*p < 0.05, ***p < 0.005—treated tumors compared to their respective, untreated, controls; ^*p* < 0.05—comparison between GFP and NCL tumors; *n* = 5). **c** Phosphorylation levels of ErbB2 (upper and lower panels) and Erk (upper panel) in dissected tumors, either untreated (upper) or treated with GroA (lower*;* NCL), were determined using anti-phospho-ErbB2 and anti-phopsho-Erk antibodies, as indicated (means ± SE; ***p < 0.005; *n* = 5)

Recently, we have reported that the nucleolar protein nucleolin triggers a ligand-independent activation of ErbB2, which appears to increase cell tumorigenicity. Moreover, high nucleolin levels in ErbB2-positive breast cancer patients correlate with poor prognosis and increased disease risk^20^. In light of this, we have used a mice xenograft model in order to determine whether overexpression of nucleolin has similar effects in vivo. For that aim, SKBR3 ErbB2-positive breast cancer cells stably expressing either GFP (SKBR3-GFP) or GFP-nucleolin (SKBR3-NCL) were injected subcutaneously into female nude mice; once tumors formed (~4 days post injection), tumor volumes were measured every 2 days. As shown, SKBR3-NCL tumors had the tendency to grow faster and were significantly larger in volume compared to SKBR3-GFP tumors (Fig. 1a, b), confirming our previous in vitro findings^20^. However, a slight decrease in volume of both types of tumors was detected at later stages (Fig. 1a); this, perhaps, due to necrosis of the tumor center, which occurred in most tumors (data not shown). Upon the termination of the experiment, ~20 days following cells injection, the tumors were dissected and tumor lysates were used to determine ErbB2 and Erk activation (phosphorylation) levels using western-blot analysis. Nucleolin overexpression has led to a significant increase in both ErbB2 and Erk phosphorylation in the tumors (Fig. 1c). Intriguingly, while the evident increase in phospho-ErbB2 levels in SKBR3-NCL tumors was usually accompanied by an increase in total ErbB2 protein levels, which was also in accordance with the results obtained previously in vitro^20^, several of the tumors exhibited a decrease in ErbB2 levels, and, on few occasions, even a complete loss of its expression.

### GroA inhibits breast cancer development in vivo

**Fig. 2:**
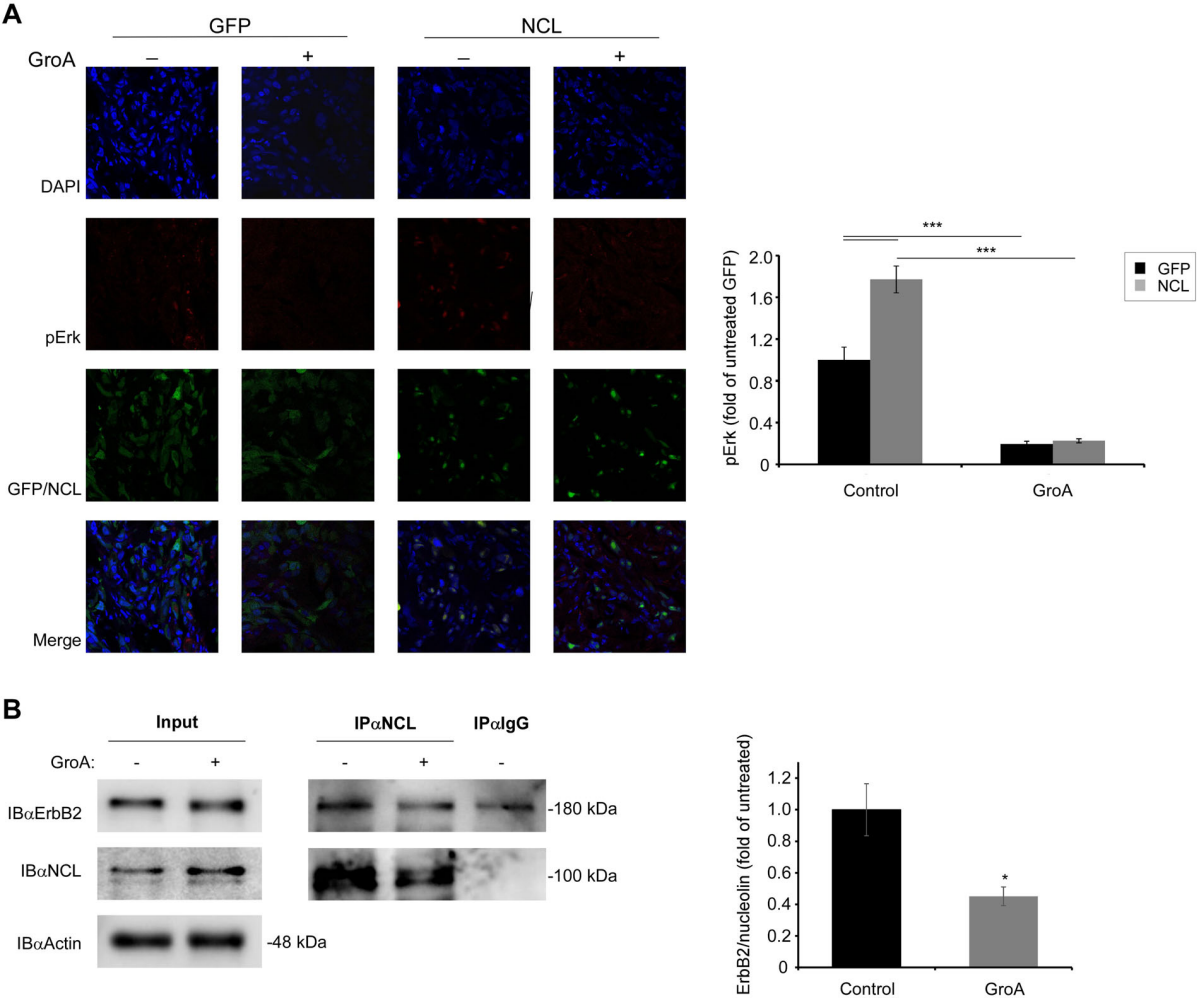
GroA reduces ErbB2-mediated signaling in vivo through disruption of ErbB2–nucleolin complexes in ErbB2-positive breast tumors **a** Phosphorylation levels of Erk in dissected GFP or NCL tumors were determined by immunostaining using anti-phopsho-Erk antibodies (means ± SE). **b** Co-immunoprecipitation (co-IP) analysis of ErbB2 and nucleolin in dissected GFP tumors following GroA treatment. Left, representative blots are presented. Right, quantification of the results (means ± SE); ErbB2 background levels, obtained from control pulldowns (right lane), were subtracted from ErbB2 levels, obtained through nucleolin pulldown (left and middle lanes); the resulting ErbB2 protein levels were normalized to nucleolin pulldown levels in each sample. *p < 0.05, ***p < 0.005, *n* = 5

Previously, we have shown that in ErbB2-positive SKBR3 breast cancer cells, inhibition of nucleolin by treatment with GroA (AS1411), a G-rich oligonucleotide^21^, reduces ErbB2 phosphorylation, and impairs cell viability and colony formation;^20^ however, cells overexpressing both ErbB2 and nucleolin were less susceptible to these effects of GroA. Since GroA was previously shown to be beneficial in terms of cancer treatment^22^ and has even been examined in phase II clinical trials for treatment of acute myeloid leukemia (AML)^23^, we sought to determine whether it had a similar effect on breast cancer in vivo. SKBR3-GFP- and SKBR3-NCL-xenografted mice, as described above, were used. Once the SKBR3-NCL tumors had reached a mean volume of ~250 mm^3^, each group was divided into two sub-groups, and administered with either GroA or vehicle treatment. Changes in tumor volumes were further monitored once every two days. As shown, both SKBR3-GFP and SKBR3-NCL tumors exhibited a significant decrease in mean volume following treatment with GroA (Fig. 1a, b). The tumors were dissected for further analysis 7–10 days after the first treatment, as described above. We found that the treatment impaired activation of both ErbB2 (Fig. 1c) and Erk (Fig. 2a), which was in accordance with the results previously obtained in cell cultures^20^.

**Fig. 3:**
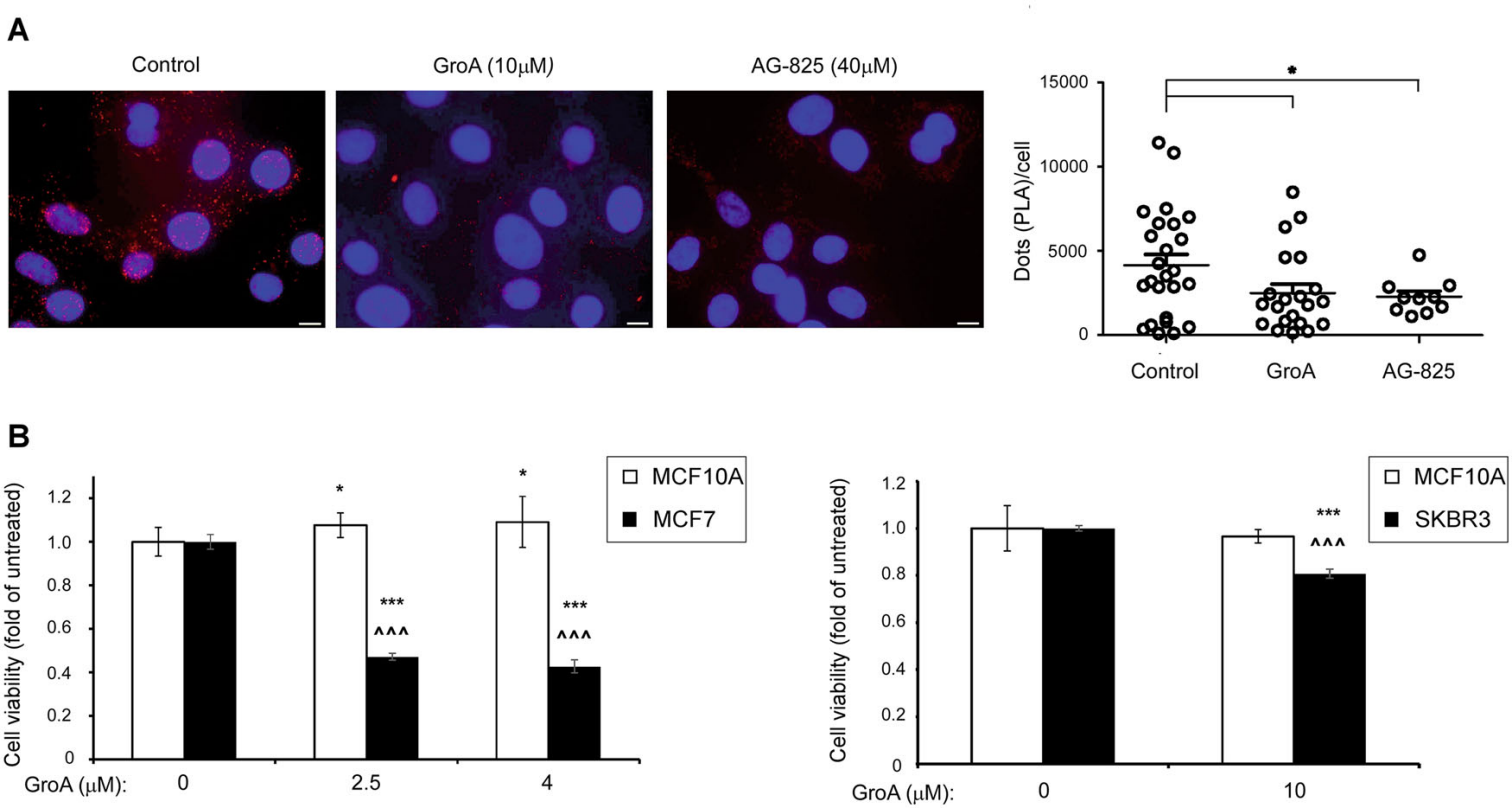
GroA and AG-825 disrupt ErbB2–nucleolin complexes, and GroA specifically inhibits viability of breast cancer cells **a** Left panel, visualization of the interaction between ErbB2 and nucleolin (red dots) in SKBR3 breast cancer cells either untreated or treated with GroA or tyrphostin AG-825, as indicated, was performed using a proximity ligation assay (PLA). Right panel, differences between signal intensity in cells represented as the number of dots per cell (means ± SE). **b** Viability of MCF10A breast cells, and of MCF7 and SKBR3 breast cancer cells following the indicated GroA treatment, as detected by methylene blue analysis (means ± SD; ***p < 0.005—untreated cells compared to treated cells of the same cell line; ^^^*p* < 0.005—comparison between MCF10A cells and MCF7/SKBR3 cells; *n* > 3)

The anti-cancer effect exerted by GroA led us to examine whether it was, in fact, caused by the disruption of the ErbB2–nucleolin interaction. As evident from a co-immunoprecipitation (co-IP) analysis of the dissected tumor xenografts (Fig. 2b), and from a proximity ligation assay (PLA), performed on SKBR3 and MDCK cells (Fig. 3a and S1A, respectively), GroA significantly reduced ErbB2 binding by nucleolin.

We next examined the specificity of GroA treatment toward cancer cells, and found that it reduces the viability of SKBR3 human breast cancer cell lines, but has virtually no effect in the non-cancerous, human breast tissue cell line, MCF10A (Fig. 3b, right). We also examined treatment sensitivity in MCF7 human breast cancer cells, which, unlike SKBR3 cells, are characterized by low ErbB2 expression^24^ and high levels of non-nucleolar nucleolin^25^. These cells were also more affected by GroA than MCF10A cells, and, not surprisingly, seeing as they are nucleolin dependent, were even more susceptible to treatment with GroA, than SKBR3 cells (Fig. 3b, left).

### Combined inhibition of nucleolin and ErbB2 enhances treatment effect

Having established that GroA has a potential as an anti-breast cancer treatment, we were interested in examining the effect of co-inhibiting both ErbB2 and nucleolin. Considering that the ErbB2–nucleolin interaction enhances the oncogenic effects of ErbB2^20^, and that inhibition of nucleolin with GroA appears to greatly affect cancer cells, we assumed that co-inhibition of both proteins may be beneficial in terms of cancer cell growth and tumorigenicity inhibition.

**Fig. 4:**
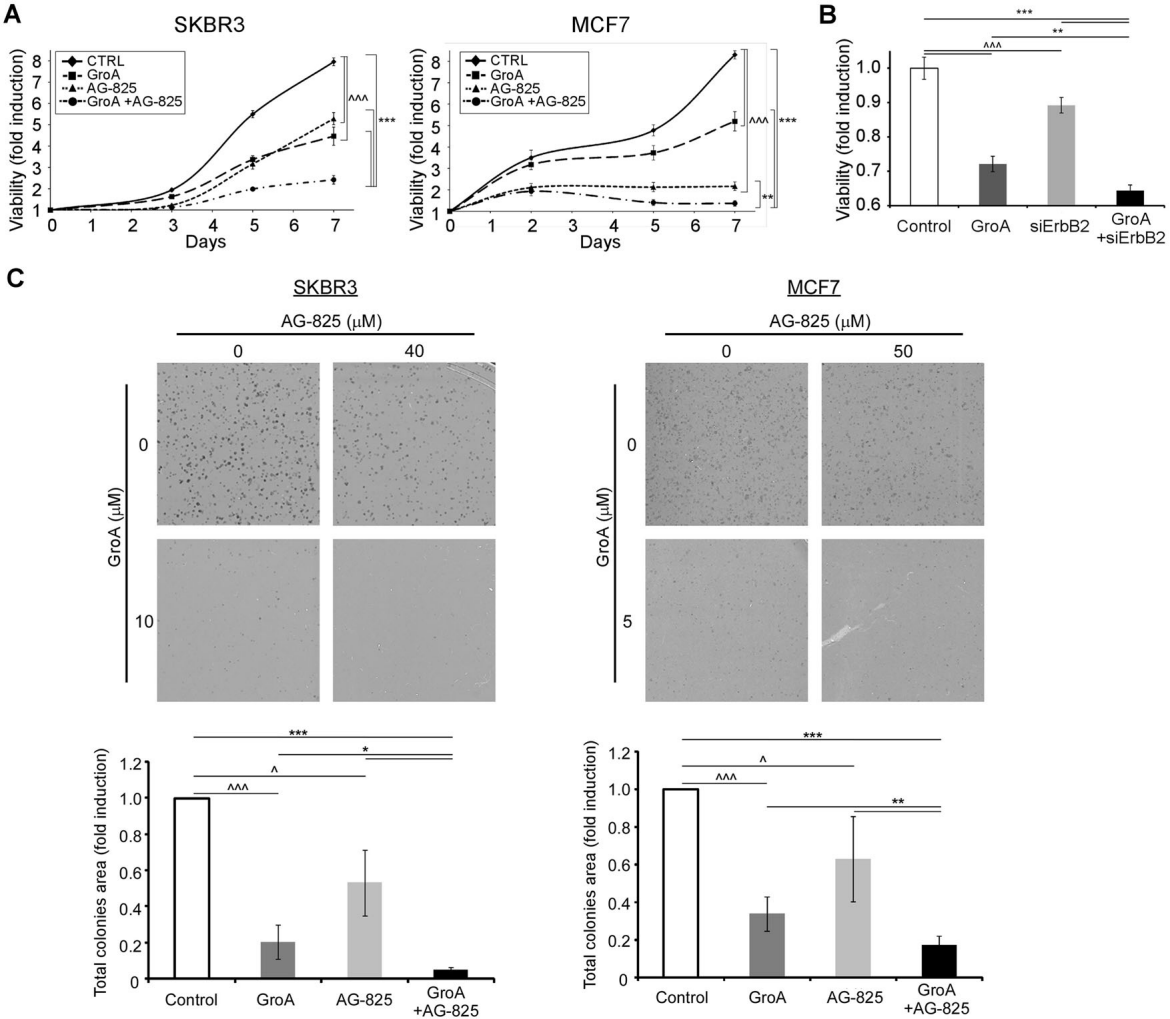
Co-inhibition of nucleolin and ErbB2 reduces breast cancer cell viability **a** SKBR3 and MCF7 cells were treated with GroA (10 and 5 μM, respectively) and AG-825 (40 or 65 μM, respectively), and cell viability was measured by the methylene blue assay at the indicated time points (means ± SD). **b** SKBR3 cells were treated with GroA (10 μM) and anti-ErbB2 siRNA, and cell viability was measured by the methylene blue assay (means ± SD). **c** SKBR3 and MCF7 cells were pre-treated with GroA and AG-825 as indicated, and total area of colonies formed was determined (means ± SD). *p < 0.05, **p < 0.01, ***p < 0.005—co-treated cells compared to untreated or single-agent treated cells; ^*p* < 0.05, ^^^*p* < 0.005—GroA/AG-825/anti-ErbB2 siRNA treated cells compared to untreated cells; *n* > 3

We have chosen to use tyrphostin AG-825, a selective ErbB2-kinase inhibitor, which we found to have a negative impact on ErbB2–nucleoin complex formation in SKBR3 and MDCK cells (Fig. 3a and S1A), for further investigation as a partner for the combined treatment. To test whether the co-treatment was more efficient than treatment with GroA alone, cell viability was analyzed in two cell lines, SKBR3 and MCF7, which were treated with GroA, AG-825, both or none for the indicated time periods (Fig. 4a). In both cell lines, the combined treatment resulted in a significant decrease in the number of viable cells, compared not only to the untreated control, but also to each treatment alone; this effect was found to be of additive nature (Fig. S1B). Similarly, co-treatment with GroA and siRNA targeted against ErbB2 significantly inhibited cell viability (Fig. 4b), indicating that the effect of GroA and AG-825 co-treatment was probably exerted by interfering with the functions of nucleolin and ErbB2, respectively. Additional experiments performed on these two cell lines indicated that similar to GroA^20^ (Fig. 4c), treatment with AG-825 also had a long-term effect on cell colony formation, as the total area of colonies was significantly smaller than that of the control. These effects were significantly augmented when the two drugs were administered concomitantly (Fig. 4c).

**Fig. 5:**
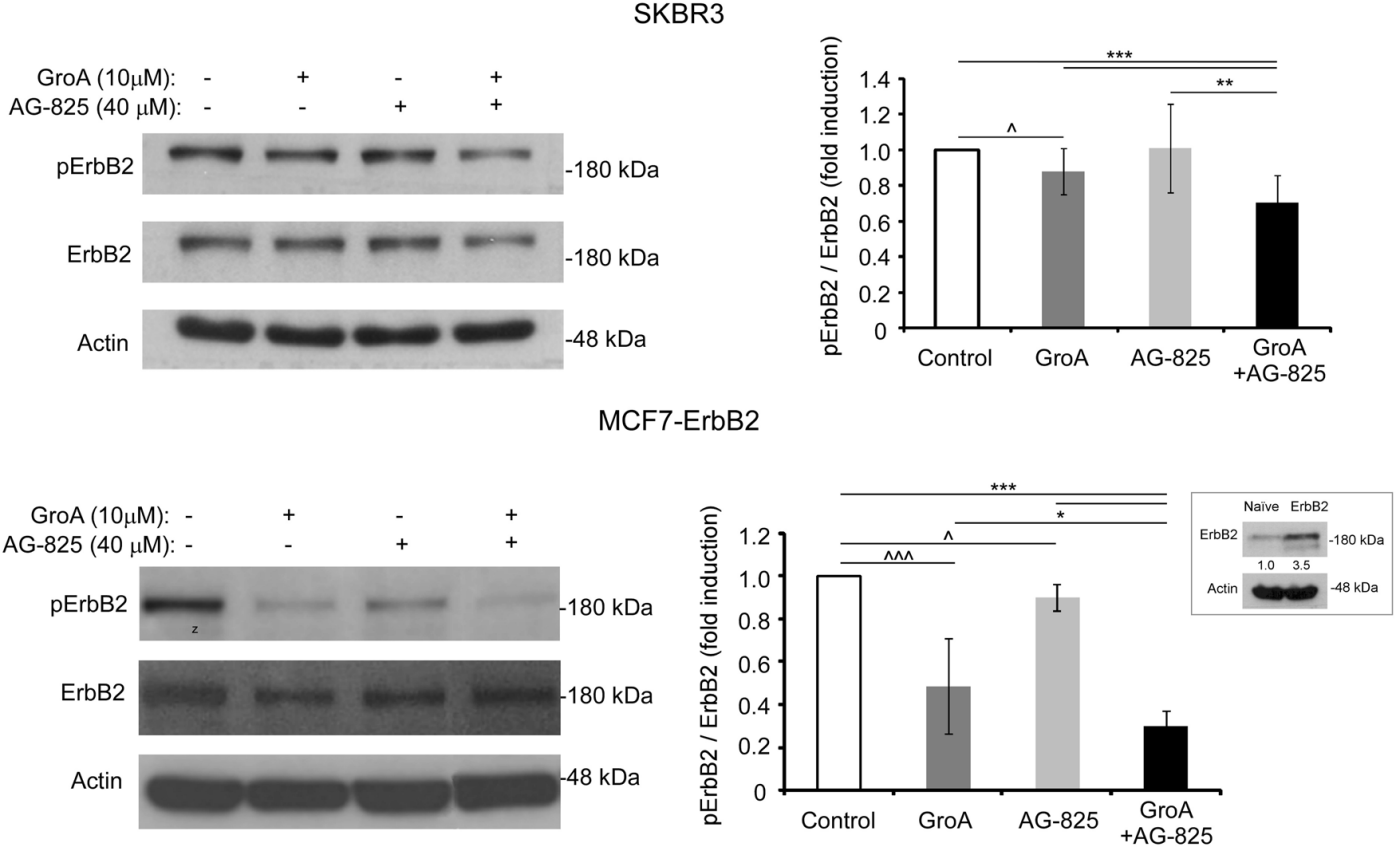
Co-treatment with GroA and AG-825 reduces ErbB2 activation SKBR3 and MCF7–ErbB2 (ErbB2-overexpressing clones) were treated with GroA, AG-825 or both, as indicated, and ErbB2 phosphorylation levels were determined using anti-phospho-ErbB2 antibody (means ± SD; *p < 0.05, **p < 0.01, ***p < 0.005—co-treated cells compared to untreated or single-agent treated cells; ^*p* < 0.05, ^^^*p* < 0.005—GroA/AG-825 treated cells compared to untreated cells; *n* > 3). Inset, immunoblot analysis of ErbB2 expression in MCF7–ErbB2 cells compared with naïve MCF7 cells; numbers below bands indicate average fold induction of naïve MCF7 cells

Next, we examined the effect of the combined treatment on receptor activation. Since endogenous levels of ErbB2 in MCF7 cells are extremely low^17^, we have used MCF7 ErbB2-overexpressing clones (MCF7–ErbB2; Fig. 5 lower panel, inset) and SKBR3 cells for western-blot analysis of ErbB2 phosphorylation levels. Notably, in both cell lines, the combined treatment had a more pronounced effect on the activation of ErbB2 than any single-drug treatment: receptor-phosphorylation levels decreased in cells treated with either AG-825 or GroA, compared to the untreated cells, and were even lower in those cells treated with the combined treatment (Fig. 5).

### The co-treatment alters cell proliferation and causes cell death in treated cells

**Fig. 6:**
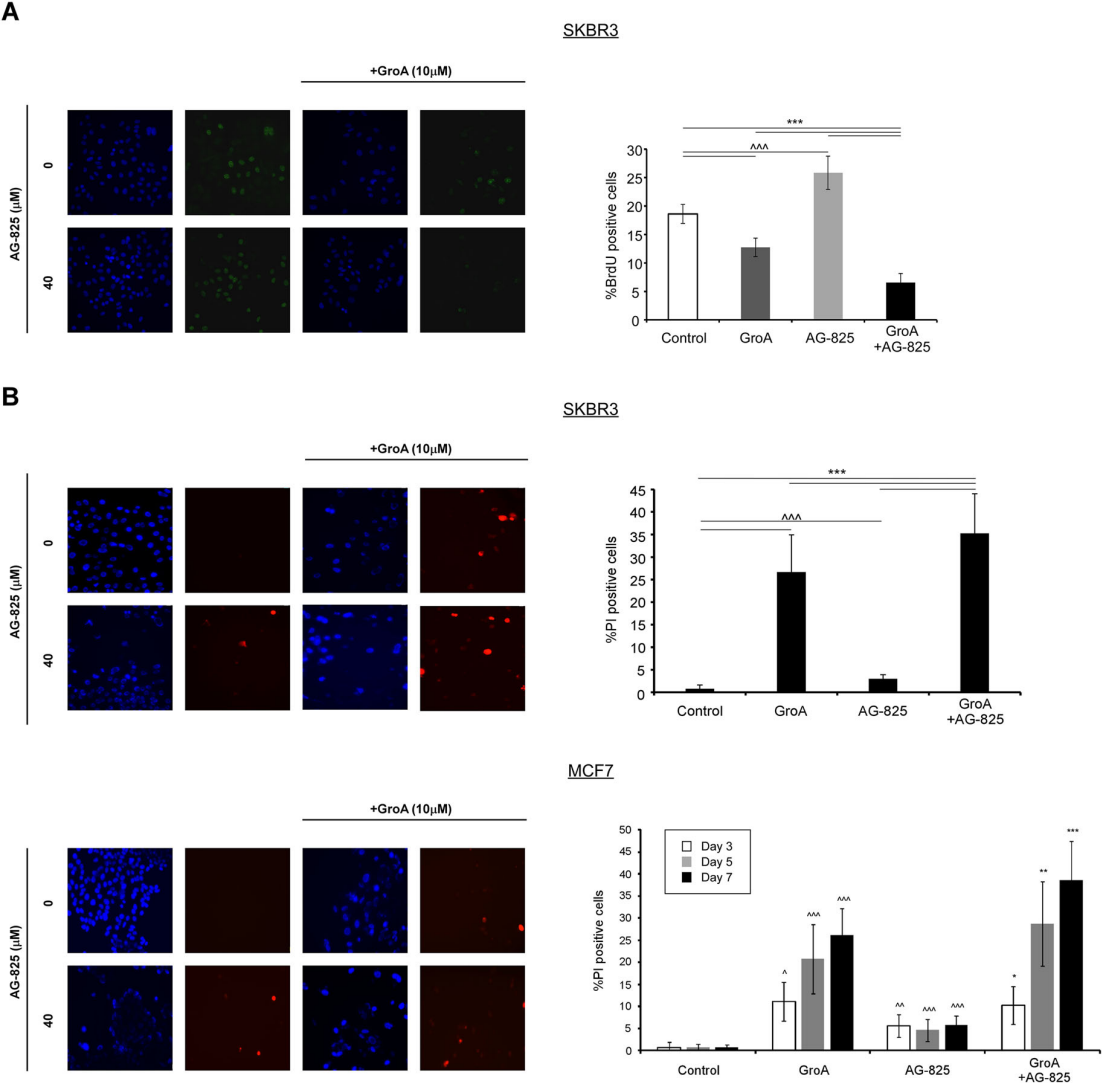
GroA and AG-825 lead to cell proliferation impairment and increased cell death **a** SKBR3 cells were treated with GroA with or without AG-825, as indicated, incubated with 5′-bromo-2′-deoxyuridine (BrdU) and subjected to immunostaining with anti-BrdU antibodies. Left panel, representative images; right panel, percentage of mitotic cells was estimated by counting the number of BrdU-positive cells compared to the number of total cells (mean ± SE). **b** SKBR3 and MCF7 cells were treated with GroA, with or without AG-825, at the indicated concentrations. The cells were stained with bisbenzimide (Hoechst) and propidium iodide (PI) to assess the number of dying cells. Left panel, representative images; right panel, percentage of dying cells was estimated by counting the number of PI-positive cells compared to the number of total cells (Hoechst-positive; mean ± S.D). *p < 0.05, **p < 0.01, ***p < 0.005—co-treated cells compared to untreated or single-agent treated cells; ^*p* < 0.05, ^^*p* < 0.01, ^^^*p* < 0.005—GroA/AG-825 treated cells compared to untreated cells; *n* > 3

Next, we have assessed the effect of GroA and AG-825 on proliferation of SKBR3 cells. Cells treated with either drug alone, none or both were subjected to the 5′-bromo-2′-deoxyuridine (BrdU) incorporation assay, and the percent of BrdU-positive cells was determined for each treatment. As shown, both GroA and AG-825 significantly altered the percentage of BrdU incorporation compared to the untreated control. While cells treated with GroA exhibited less BrdU staining, in cells treated with AG-825 the rate of BrdU-positive cells was significantly greater (Fig. 6a). These results suggest different replication alterations caused by each of the drugs. Interestingly, co-treatment with both drugs led to a reduction in BrdU-positive cells, which was significantly greater than the reduction caused by GroA alone. Since each agent affected the cells differently, we assumed that the combined negative effect on BrdU staining could be a result of increased cell death. Therefore, in order to test whether the reduction in cell growth of co-treated cells stemmed from cell death, a dye exclusion assay was performed in SKBR3 and MCF7 cell lines. This has, indeed, revealed that cell death was significantly enhanced by the co-treatment with GroA and AG-825 compared to the single-drug treatments (Fig. 6b).

### The combined treatment impairs cell tumorigenicity

**Fig. 7:**
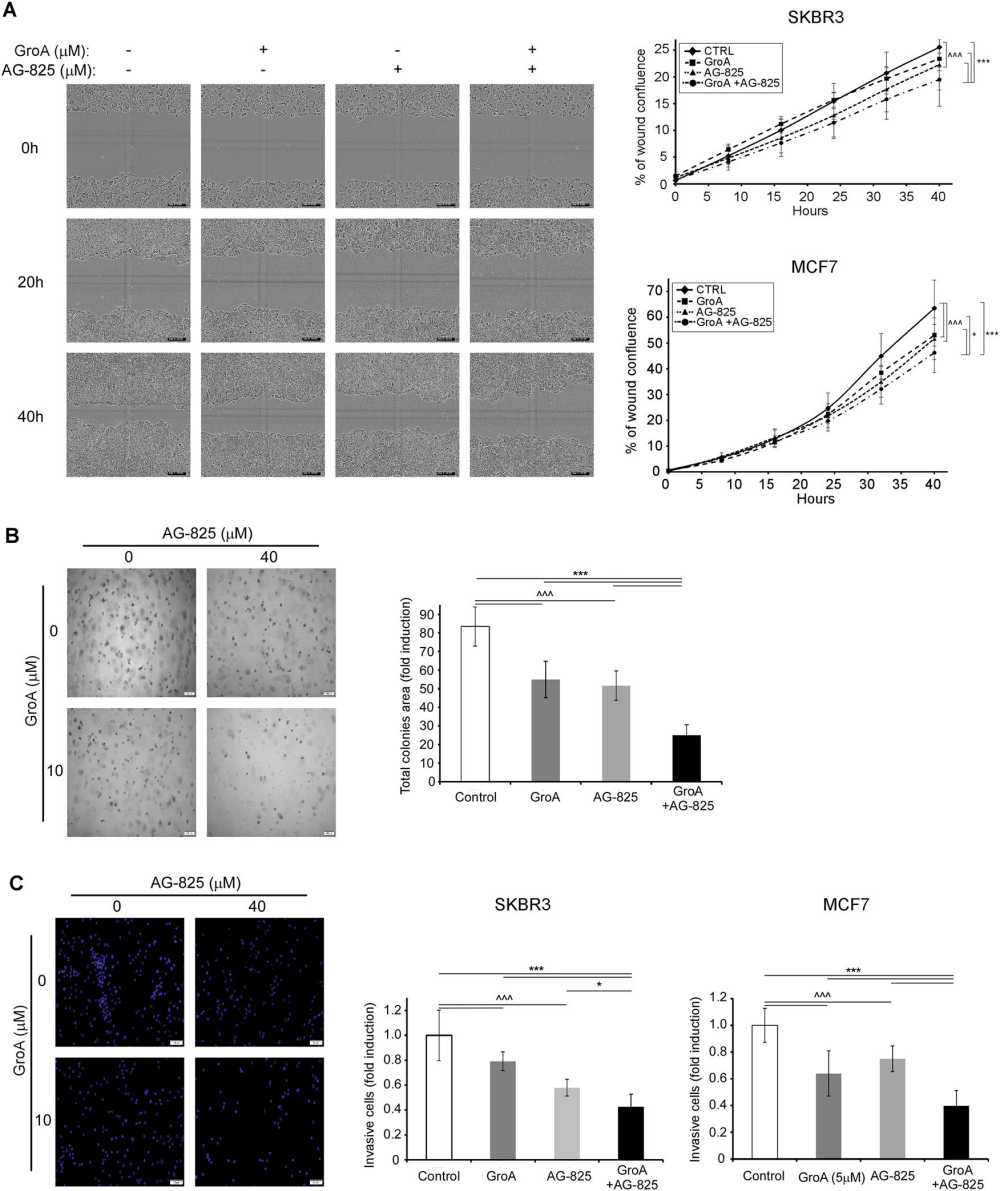
Combined treatment with GroA and AG-825 impairs breast cancer cell tumorigenicity **a** Migration rate of SKBR3 and MCF7 cells in the presence of GroA, AG-825 or both was determined using the scratch assay. Left panel, representative images of SKBR3 cells 0, 20 and 40 h post-wound infliction; right panel, cell migration rate during 40 h post-wound infliction, represented as percent of wound confluence (results from representative experiments are shown; means ± SD; *n* > 3). **b** SKBR3 cells ability to grow in an anchorage-independent manner in the presence of GroA, AG-825 or both was examined using the 3D basement membrane culture assay. **c** SKBR3 and MCF7 cells were co-treated with GroA and AG-825, and subjected to cell invasion analysis. Left panel, representative images of SKBR3 cells; middle and right panels, number of cells that successfully penetrated the Cultrex basement membrane layer (means ± SD). *p < 0.05, ***p < 0.005—co-treated cells compared to untreated or single-agent treated cells; ^^^*p* < 0.005—GroA/AG-825 treated cells compared to untreated cells; *n* > 3

We have next tested the effect of the co-treatment on cell tumorigenicity by assessing the metastatic potential of the cells. First, we have examined stress fibers formation following the co-treatment by staining the actin cytoskeleton of SKBR3 cells. The obtained results revealed that the amount of stress fibers in cells treated with either drug alone was higher, compared to untreated cells, and further increased in cells receiving the combined treatment (Fig. S1C), suggesting cell migration impairment. We then tested cell migration by performing a scratch assay in SKBR3 and MCF7 cells treated with each drug alone or both for 40 h. Cells co-treated with GroA and AG-825 migrated significantly slower than the untreated control cells or cells treated with each drug alone (Fig. 7a). Consistently, the effect of GroA was similar to that of RNA interference against nucleolin, indicating specificity of its effect (Fig. S2A).

In order to further establish the effect of GroA and AG-825 treatment on cell tumorigenicity, we tested SKBR3 growth in a three-dimensional culture. This culturing method allows the investigation of both the anchorage-independent growth ability of the cells and the level of culture organization^26,27^. Our results indicate that both GroA and AG-825 interfered with the ability of SKBR3 cells to grow in an anchorage-independent manner, and that this effect was more prominent in the co-treated cells; however, no changes in growth morphology following any treatment were observed (Fig. 7b).

Finally, since migration and anchorage-independent growth, solely are not sufficient for efficient metastasis, which also requires penetration of the basement membrane^28^, the invasiveness of SKBR3 and MCF7 cells in the presence of the co-treatment was examined. Treatment with both GroA and AG-825 significantly diminished the number of cells that were able to invade through Cultrex basement membrane, compared to each drug alone and the untreated control (Fig. 7c). In this setting, as well, GroA seemed to exert an effect that was similar to that of nucleolin-targeted shRNA (Fig. S2B).

## Discussion

Nucleolin and ErbB2 are key components of the cell’s survival, growth, and proliferation machinery^1,13,19^. Both proteins are tightly linked to cell transformation and are known oncogenes, with ErbB2 overexpression being one of the major causes of breast cancer^4,29,30^. Breast cancer is one of the most common malignancies, and is a leading cause of cancer-associated deaths among women worldwide^31^. ErbB2-positive breast cancer is associated with higher mortality rates and multi-drug resistance^5,32^. Recently, we have found that ErbB2 and nucleolin physically interact in cells, and that this interaction leads to activation of ErbB2 and its downstream signaling, which culminates in increased cell growth and tumorigenicity, and in increased disease and mortality risk in breast cancer patients^6,20,33^. Moreover, we have shown that these oncogenic traits can be mitigated in cancer cells through competitive nucleolin binding by a truncated ErbB1 variant, and by specific inhibition of nucleolin using RNAi and the GroA (AS1411) G-rich oligonucleotide^20^. Cells treated by GroA were found to exhibit a significant decrease in viability, which was accompanied by a reduction in ErbB2 phosphorylation (activation) levels. Furthermore, the susceptibility of SKBR3 cells to GroA treatment appeared to depend upon nucleolin levels. Indeed, our present in vivo experiments performed on ErbB2-positive breast cancer xenograft mice seemed to corroborate our previous findings in the SKBR3 cancer cell line. Since this cell line has been previously used in vivo by several studies^34,35^, we chose to use it in our breast cancer xenograft model. Injected cells readily formed tumors, which rapidly increased in size. Tumors overexpressing nucleolin appeared to be more aggressive than tumors with normal endogenous nucleolin levels; however, both types of tumors also exhibited a later decrease in volume, which was attributed to necrosis of their inner tissue (data not shown). In this regard, it should be noted that, in the past, some reports have deemed SKBR3 cells poorly tumorigenic in vivo^36^. This could be a result of the observed rapid tumor formation, which does not allow for proper tumor vascularization, and the consequent death of the cells in its center. Nevertheless, additional decrease in tumor volume after its initial reduction was not detected, and, in some cases, it was even followed by renewed tumor growth (data not shown). Interestingly, both types of tumors reacted to treatment with GroA, following which, the tumors have shrunken, and activation of ErbB2 and Erk was decreased. Since we have also observed a concomitant decrease in nucleolin binding to ErbB2 both in vitro and in vivo, it is plausible to assume that the anti-cancer effects of GroA were mediated by the disruption of ErbB2–nucleolin complexes. Notably, treatment with GroA was found to affect breast cancer, but not normal, cell lines, indicating treatment specificity toward malignant cells.

Acquired resistance to anti-ErbB2-targeted drugs is relatively common^37^, and poses a major challenge in treatment of ErbB2-positive breast cancer. For this reason, we assumed that targeting of the ErbB2–nucleolin interaction by anti-cancer drugs could represent a potential novel approach to breast cancer therapy. The results obtained with GroA prompted us to examine its effect in combination with ErbB2 inhibition on cancer cell growth, in an attempt to improve treatment outcome. We found that tyrphostin AG-825, a specific ErbB2-kinase inhibitor, leads to a decrease in ErbB2–nucleolin interaction, and since it was previously found to affect tumorigenicity of several types of ErbB2-positive cancers, including breast cancer^34–36,38^, we chose this inhibitor for further study in combination with GroA. Indeed, co-treatment with both agents resulted in an additional, significant, decrease in ErbB2 phosphorylation, accompanied by a reduction in cell viability and colony formation in SKBR3 and MCF7 cells, compared to each drug alone. This impairment of cell viability was analogous to the decrease in viability obtained when cells were treated with a combination of GroA and an anti-ErbB2 siRNA, suggesting that the effect of the AG 825-GroA co-treatment was mediated by interference with the nucleolin–ErbB2 interaction.

Furthermore, this co-treatment led to defective cell proliferation and increased cell death, as was evident by BrdU incorporation and dye exclusion assays; however, BrdU staining of treated cells has rendered rather different results for both inhibitors. While the percent of BrdU-positive cells decreased in the presence of GroA, it was enhanced when cells were treated with AG-825. This could be a result of differential disruption of cell proliferation. According to previous reports, GroA causes an S-phase arrest^39^; early S-phase arrest would result in a reduction of BrdU incorporation, as DNA replication is initiated in fewer cells. The increase in BrdU staining caused by the presence of AG-825, on the other hand, might indicate an impairment of later cell cycle stages, i.e., the cells are unable to proceed in their division following the S-phase. Interestingly, despite this, cells treated with the combination exhibited an additional, significant, decrease in percent of BrdU-stained cells, compared to untreated cells and treatment with GroA alone. This might be due to the enhanced cell death caused by the co-treatment, which led to a depletion of BrdU-positive cells from the culture. As GroA and AG-825 both inhibit proteins that are key participants in cell growth and proliferation^19,40^, such results are in accordance with the changes observed in ErbB2 signaling following treatment with GroA alone and in combination with AG-825.

Overall, cell tumorigenicity seemed also to decline as a function of the administered treatment; cells that received the combined treatment exhibited slower migration than cells treated with either GroA or AG-825 alone. The altered migration was accompanied by impaired anchorage-independent growth and invasion of the cells, thus suggesting a decrease in the cells’ metastatic ability^26^.

In summary, our data indicate that GroA might prove to be an efficient agent in the treatment of breast cancer, including ErbB2-positive tumors. The incidence of breast cancer-related mortality is high, especially in ErbB2-positive patients, and acquired resistance to ErbB2-targeted anti-cancer drugs constitutes a major setback for treatment. Therefore, identification of novel targets for therapy, which could interfere with ErbB2 signaling in a better, enhanced, manner, is important. The ability of GroA to prevent ErbB2 activation, despite not having a direct effect on the receptor, suggests that GroA can be used as a therapeutic solution in cases of acquired resistance to ErbB2-targeted drugs. Furthermore, combined targeting of nucleolin and ErbB2 in breast cancer cells appears to be beneficial in terms of anti-cancer therapy. Co-treatment with GroA and AG-825 had a more profound effect in each of the parameters tested in two breast cancer cell lines. Since both cell lines have different genomic profiles, as SKBR3 cells endogenously express high levels of ErbB2, whereas its expression in naïve MCF7 cells is low, it is possible that the effect of the combined treatment is indeed mediated by disruption of the nucleolin–ErbB2 interaction. Although AG-825 is not applicable in vivo, as the compound lacks sufficient stability in live tissues^41^, the impact of its combination with GroA on cancer cells constitutes a proof of concept, and it can be later replaced by other ErbB2-inhibiting agents and clinical drugs, such as Herceptin^42,43^. Further research on the clinical relevance of interference with ErbB2–nucleolin complex formation is required, as targeting it with AG-825 and GroA proved to be effective.

## Materials and methods

### Materials and buffers

The antibodies used are as follows: monoclonal mouse anti-actin (691001; MP Biomedicals, Santa Ana, CA); polyclonal rabbit anti-ErbB2 (HER2/neu), and monoclonal mouse anti-GFP (sc-284 and sc-9996, respectively; Santa Cruz Biotechnology, Dallas, TX); polyclonal rabbit anti-phospho-ErbB2 (2249; Cell Signaling Technology, Danvers, MA); polyclonal rabbit anti-GFP (632460; Clontech); and monoclonal mouse anti-BrdU (11170376001; Roche).

Tyrphostin AG-825 (10010243) was from Cayman Chemical. The aptamer GroA (AS1411) and the inactive control oligomer Cro, were purchased from IDT (Jerusalem, Israel) as unmodified desalted oligonucleotides, as previously described^44^.

### Cell lines

Human breast cancer cell lines SKBR3 and MCF7 were all grown in Dulbecco’s modified Eagle’s medium (DMEM; Biological Industries, Beithaemek, Israel). All media were supplemented with antibiotics and 10% heat-inactivated fetal bovine serum (FBS; Hyclone, Thermo Scientific, Waltham, MA). Cells were incubated at 37 °C in 5% CO_2_ in air, and the medium was changed every 3‒4 days. When 70% confluent, cells were passaged in trypsin/disodium ethylenediaminetetraacetic acid (Biological Industries, Beithaemek, Israel). One day before treatment, the cells were plated at ~50% confluence in medium supplemented with 10% fetal calf serum. Concentrations for GroA treatments (control treatments with Cro oligonucleotide) and AG-825 treatments (control treatments with 1% DMSO), as well as the duration of treatment, where relevant, are indicated for each experiment.

### DNA and siRNA transfections

Generation of SKBR3-GFP and SKBR3-NCL (overexpressing either GFP or GFP-nucleolin, respectively) was as previously described^20^. MCF7 cells overexpressing ErbB2 (MCF7–ErbB2) were a gift from Prof. Y. Yarden, Weizmann Institute of Science, Israel.

Anti-ErbB2 siRNA and AllStars Negative Control siRNA (SI04948811; 1027280, respectively; QIAGEN) were transfected using the HiPerFect Transfection Reagent (301704; QIAGEN) according to the manufacturer’s instructions. Cells were subjected for further analysis 72 h post transfection.

### In vivo tumor xenograft studies

The study was conducted according to the NIH Guidelines for Use and Care of Laboratory Animals and following approval by the Animal Care Committee of the Tel Aviv University, no. 14-15-041

Female, 8-week old, athymic nude mice (Foxn1^nu^; Harlan) were injected subcutaneously with ~7.5 × 10^6^ SKBR3-GFP or SKBR3-NCL cells in 100 µl 40% Matrigel (BD Bioscience; 356234) in PBS. Formation and growth of tumors were monitored every 2 days. Once the tumors reached ~250 mm^3^ in size, the mice were divided into four groups according to xenograft type (SKBR3-GFP/NCL) and treatment (control/GroA); treatment was administered as previously described^45^, and tumor volumes were measured every 2 days.

Upon the end of the experiment (~20 days post cells injection), the mice were sacrificed, and the tumors were dissected and used for further analysis. For western-blot analysis, tumors were homogenized in solubilization buffer using a polytron homogenizer and processed as described below under “Lysate preparation and immunoprecipitation”; for immunostaining, tumors were fixed in 4% PFA (paraformaldehyde), incubated in 20% sucrose in PBS overnight at 4 °C, and cut Cryostat sections (20 µm) were further mounted on slides and fixed in 4% PFA. Next, sections were incubated in blocking solution followed by incubation with primary antibodies diluted in blocking solution. After washing, sections were incubated at room temperature with secondary antibodies diluted in blocking solution, nuclei were stained with DAPI (1 µg/ml; Sigma-Aldrich), and the sections were mounted in Fluoromount (Dako). Finally, the sections were examined using a Leica TCS SP8 confocal microscope (×63 magnification).

### Proximity ligation assay

For PLA cells were plated in 16-well Nunc Lab-Tek glass Chamber Slide System (178599; Thermo Scientific) and treated as indicated for 2 days. Following fixation, cells were incubated with rabbit anti-ErbB2 and mouse anti-nucleolin antibodies. PLA was performed using the Duolink In-Situ PLA probes: anti-rabbit MINUS and anti-mouse PLUS, and the Duolink In-Situ Detection Reagents Red kit (DUO92005; DUO92001; DUO92008, respectively; Sigma-Aldrich), according to the manufacturer’s instructions. Nuclei were stained using the Duolink In-Situ Mounting Medium with DAPI (DUO82040; Sigma-Aldrich). Slides were visualized 24 h post staining and images were obtained using an Olympus motorized inverted research microscope Model IX81 (×60 magnification). Signal intensity was determined using ImageJ software.

### Methylene blue viability assay

SKBR3 and MCF7 cells were plated in medium supplemented with 10% FBS and treated as indicated for the different experiments, and cell numbers were determined at the indicated times. For this purpose, the cells were fixed with 4% formaldehyde in PBS and incubated with the DNA-binding dye methylene blue (1% in boric acid) at room temperature. The cells were then lysed with 0.1 M HCl. Absorbance was measured with a Tecan Spectrafluor Plus spectrophotometer (Mannedorf, Switzerland) at 595 nm. Cell viability was calculated as the ratio of absorbance in treated cultures to that in untreated control cultures 1 day after seeding.

### Colony formation (clonogenic) assay

SKBR3 cells were plated onto six-well plate and treated on the following day as indicated. After treatment, the cells were detached and replated on 10-cm plates (1:10, 1:20, 1:40 dilutions). The cells were fixed with 0.1% acetic acid in PBS 7–11 days later, respectively, and then stained with 0.4% crystal violet in acetic acid. Total colonies area was calculated using the ImageJ program.

### Lysate preparation and immunoprecipitation

After the indicated treatment, cells were lysed in solubilization buffer. Lysates were cleared by centrifugation, sample buffer was added and the samples were boiled. For immunoprecipitation assays, antibodies were coupled to anti-IgG agarose beads. The beads were then incubated with cell lysates. The immunoprecipitates were washed and the proteins were eluted by addition of sample buffer and boiling. For all immunoblotting, proteins were resolved by SDS-polyacrylamide gel electrophoresis through 10‒12.5% polyacrylamide gels, and were electrophoretically transferred to nitrocellulose membranes. Membranes were blocked in TBST buffer containing 6% milk, and blotted with primary antibodies. Secondary antibody linked to horseradish peroxidase was then added. Immunoreactive bands were detected with the enhanced chemiluminescence reagent.

### Dye exclusion assay

Cells were plated in medium supplemented with 10% FBS and treated as indicated for 5–6 days, depending on cell line. To estimate the number of dead cells, live cultures were incubated for 10 min with the membrane-permeable fluorescent DNA dye bisbenzimide (Hoechst 33342, 1 µg/ml; Sigma-Aldrich) and the membrane-impermeable fluorescent DNA dye propidium iodide (PI, 1.5 µg/ml; Sigma-Alrdich). After staining, the cells were photographed with an Olympus motorized inverted research microscope Model IX81 (×20 magnification). The percentage of dead cells was estimated by calculating the number of PI-stained nuclei relative to the total, Hoechst 33342-stained nuclei, in each field, from over 30 random fields.

### BrdU immunostaining

Cells were seeded on coverslips coated with poly-l-Lysine in medium supplemented with 10% FBS. The cells were then pre-treated as indicated for 4 days. For BrdU staining, the cells were incubated with 5′-bromo-2′-deoxyuridine (BrdU, 50 µM; Sigma-Aldrich) for 2 h. Next, cells were fixed with 4% PFA followed by incubation in blocking solution. Primary anti-BrdU antibody was diluted in blocking solution. After washing, cells were incubated at room temperature with secondary antibodies diluted in blocking solution. Cell nuclei were stained using bisbenzimide (Hoechst 33258, 1 µg/ml; Sigma-Aldrich), and the cells were mounted in Fluoromount (Dako). Cells were examined under a fluorescence microscope at ×20 magnification with Olympus motorized inverted research microscope. The percentage of proliferating cells was estimated by calculating the number of BrdU-positive cells relative to the total, Hoechst 33258-stained, number of cells, from over 30 random fields.

### Scratch-induced migration assay

Cells were plated at high confluence in medium supplemented with 10% FBS in 96× well IncuCyte ImageLock Plates (Essen BioScience; 4379). The following day, the WoundMaker-IncuCyte ZOOM-ImageLock Plate system was used to inflict a scratch wound and the cells were treated as indicated. Imaging and calculation of the wound gap was obtained every 2 h for the indicated time periods, using the IncuCyte ZOOM Live-Cell Analysis System (Essen BioScience; http://www.essenbioscience.com/media/uploads/files/8000-0195-A00_ZOOM_Scratch_Wound_Tech_Note.pdf).

### 3D basement membrane culture assay

The 3D basement membrane culture assay was performed according to the method previously described by Lee et al.^17^, with slight modifications. Wells were pre-coated with 25 µl of Cultrex BME (Trevigen; 3432-005-01); once the coating has gelled, cells were resuspended in Cultrex BME and transferred to the coated wells (55 µl/well). Medium supplemented with 10% FBS and containing the indicated treatment was added on top of the embedded cells. Cells were then allowed to grow for 8–9 days; treatments were refreshed every 3–4 days. Images were obtained using an Olympus motorized inverted research microscope Model IX81 (×4 magnification), and quantified using the ImageJ software.

### Cell invasion assay

The assay was performed according to the method previously described by Zeng et al.^27^, with slight modifications. Prior to seeding of cells, 6.5 mm Transwell (8.0 µm pores; Corning; 3422) plates were pre-coated with poly-l-lysine. Cultrex BME (Trevigen; 3432-005-01) coating of upper chambers was performed as previously described, and followed by addition of cells resuspended in 100 µl of 50% Cultrex BME in starvation medium containing the indicated treatments; medium supplemented with 10% FBS and containing the respective treatments was added to the lower chambers. The cells were allowed to migrate for 24 h, then the upper chamber was fixed in 4% formaldehyde in PBS and stained with the DNA-binding fluorescent dye bisbenzimide (Hoechst 33258, 1 µg/ml; Sigma-Aldrich). Images were obtained using an Olympus motorized inverted research microscope Model IX81 (×10 magnification), and quantified using the ImageJ software.

### Statistical analysis

All experiments were performed at least three times. Results are presented as means ± SD/means ± SE. Differences between means were assessed by the one-tailed Student’s *t* test, one-way ANOVA, two-way ANOVA, and ANCOVA tests using GraphPad Prism v. 5.03 for Windows and JMP v. 12.0.1 softwares. Significance was assigned at *p* < 0.05.

## Electronic supplementary material

Supplementary

Supplementary Figure 1

Supplementary Figure 2
