# Supplementary material for: Nucleolin and ErbB2 inhibition reduces tumorigenicity of ErbB2-positive breast cancer
Source: Cell Death Dis. 2018 Jan 19;9(2):47. doi: 10.1038/s41419-017-0067-7 (PMC5833446; doi:10.1038/s41419-017-0067-7)
Supplement: Supplementary file 1 — Supplementary [file 41419_2017_67_MOESM1_ESM.docx]

**Supplementary figure legends**

**Figure S1: Treatment with GroA and AG-825 disrupts ErbB2-nucleolin complexes, is additive in nature, and leads to stress fibers formation.** (A) Differences between number of ErbB2-nucleolin complexes in MDCK cells following treatment with either GroA or AG-825, as detected by PLA (signal intensity represented as the number of dots per cell; means ±SE). (B) Analysis of the additive effect of GroA and AG-825 combination. SKBR3 cells were treated for 5 days, using increasing concentrations of the drugs, either alone or in combination. *Upper panel,* cell viability was tested using the methylene blue staining assay. Results are presented as fold induction over the control untreated cells (means ±SD). *Lower panel,* the combination index was calculated as described in supplementary Materials and Methods and is plotted vs. the affected cells fraction. (C) Stress fibers in SKBR3 cells, treated with GroA, AG-825 or both as indicated, that were subjected to immunostaining using the actin-binding fluorescent dye phalloidin-rhodamine.

**Figure S2: Depletion of nucleolin impairs breast cancer cell migration and invasiveness.** (A) Migration rate of SKBR3 cells subjected to treatment with either mock or anti-nucleolin shRNA was determined using the scratch assay. *Left panel*, cell migration rate during 40h post-wound infliction, represented as percent of wound confluence; *right panel,* representative images of SKBR3 cells 0, 20 and 40h post-wound infliction (results from representative experiments are shown; means ±SD; n>3). (B) SKBR3 cells were treated with either mock or anti-nucleolin shRNA, and subjected to cell invasion analysis. *Left panel*, number of cells that successfully penetrated the Cultrex basement membrane layer (means ±SD); *right panel*, representative images.

**Supplementary Materials and Methods**

**Cell lines**

MDCK cells were cultured in Dulbecco's modified Eagle's medium (DMEM; Biological Industries, Beithaemek, Israel), as described in Materials and Methods.

**Analysis of the cytotoxic effect of GroA in combination with AG-825**

The effect of drug combination was calculated according to the median effect principle, described by Chou and Talalay (1984). First, we constructed the dose-response curves for the cytotoxic effects of GroA and AG-825 alone, and in combination, in SKBR3 cells, using the methylene blue staining assay. The data was used to determine the ‘combination index’ (CI), using the equation: CI = (D)1/(Dx)1+(D)2/(Dx)2, where (D)1 and (D)2 are the combinations doses that kill x% of cells, and (Dx)1 and (Dx)2 are the doses of each drug alone that kill x% of cells. Synergism is assigned at CI<1, whereas CI≈1 indicates an additive effect.

**shRNA transfections**

Anti-nucleolin shRNA (62-320; Upstate) and AllStars Negative Control siRNA (1027280; QIAGEN) were transfected using the HiPerFect Transfection Reagent, as described in Materials and Methods.

**Phalloidine-rhodamine immunostaining**

Cells were seeded on coverslips coated with poly-L-Lysine in medium supplemented with 10% FBS. The cells were then treated as indicated for 4 days, fixed and permeabilized as described in Materials and Methods. Next, cells were stained with phalloidin–tetramethylrhodamine B isothiocyanate (phalloidin, 1µg/ml, Sigma-Aldrich), mounted in Fluoromount (Dako), and nuclei were stained using DAPI (1µg/ml; Sigma-Aldrich). Cells were examined using a Leica TCS SP8 confocal microscope (×63 magnification).
