# Supplementary figures and images for: Nucleolin and ErbB2 inhibition reduces tumorigenicity of ErbB2-positive breast cancer
Source: Cell Death Dis. 2018 Jan 19;9(2):47. doi: 10.1038/s41419-017-0067-7 (PMC5833446; doi:10.1038/s41419-017-0067-7)

**A**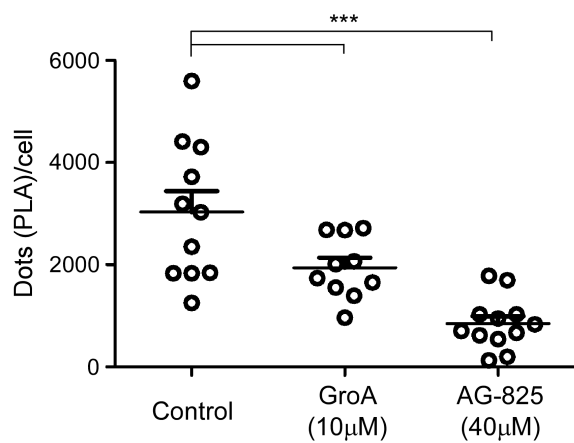**B**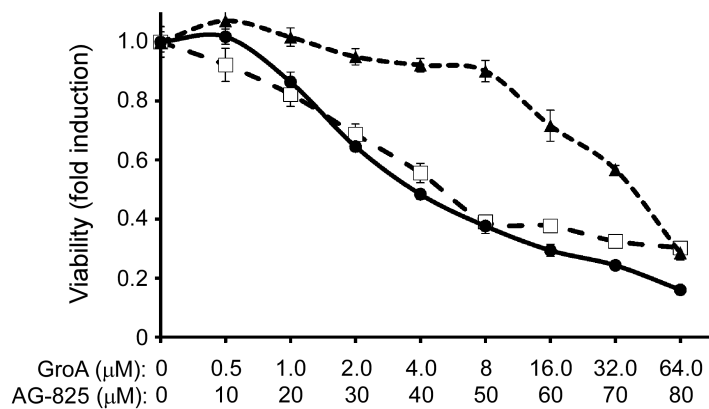**C**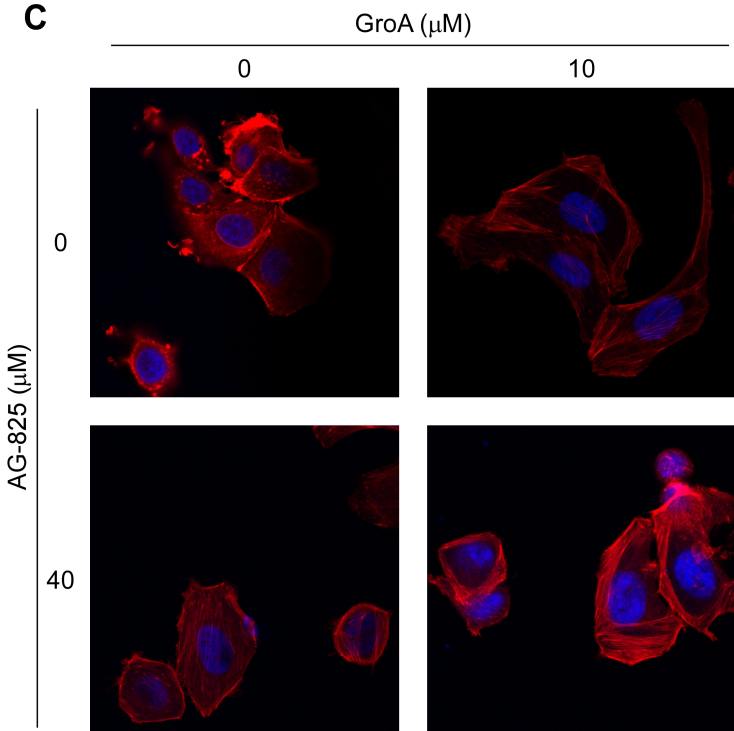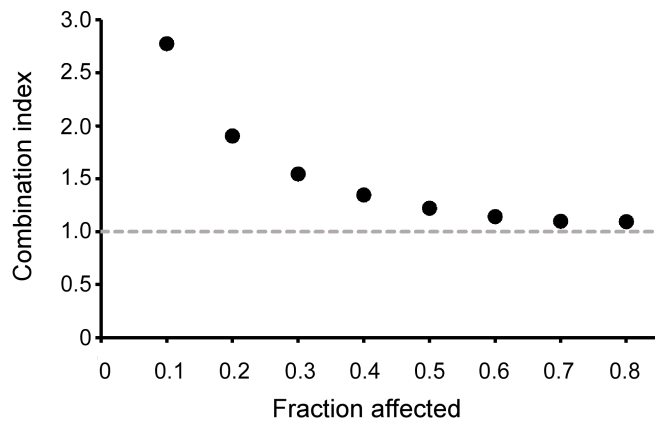

Supplement: Supplementary file 2 — Supplementary Figure 1 [file 41419_2017_67_MOESM2_ESM.pdf]

**A**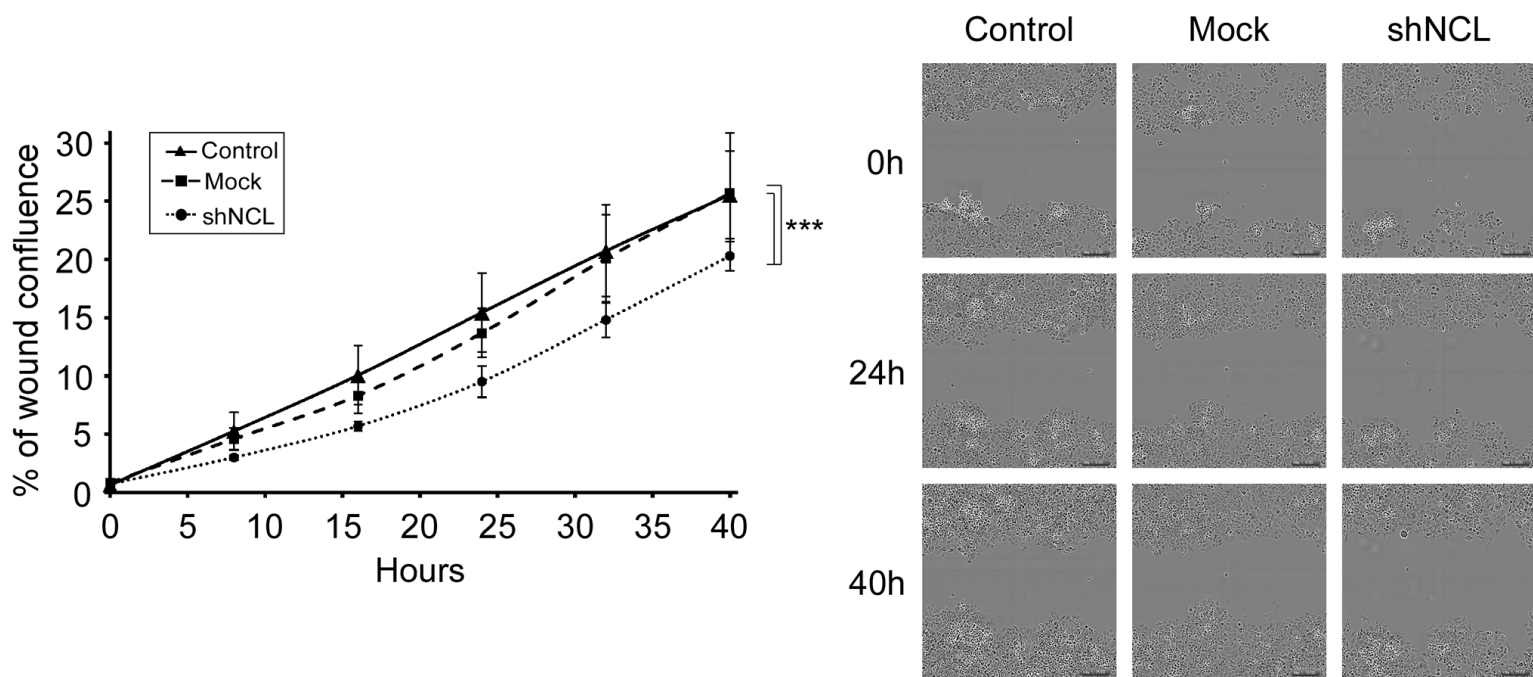**B**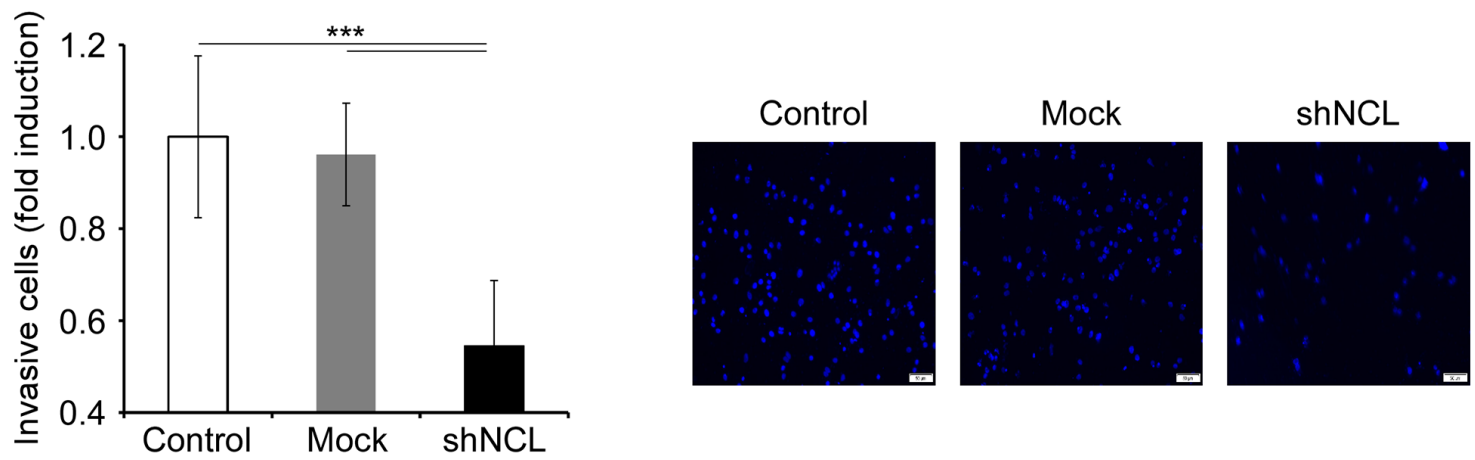

Supplement: Supplementary file 3 — Supplementary Figure 2 [file 41419_2017_67_MOESM3_ESM.pdf]
